# Microstructure and microhardness of molybdenum processed by monotonic and cyclic high-pressure torsion

**DOI:** 10.1007/s10853-026-13384-6

**Published:** 2026-08-10

**Authors:** Saleh N. Alhajeri, Khaled J. Al-Fadhalah, Abdulla I. Almazrouee, Fahad S. Alhajri, Yi Huang, Terence G. Langdon

**Affiliations:** 1 Department of Manufacturing Engineering, College of Technological Studies, PAAET, P.O. Box 42325, 70654 Shuwaikh, Kuwait; 2 Department of Mechanical Engineering, College of Engineering and Petroleum, Kuwait University, Sabah Al Salem University City, P.O. Box 5969, 13060 Safat, Shadadiya, Kuwait; 3 Department of Mechanical Production, Vocational Training Institute, PAAET, P.O. Box 42325, 70654 Shuwaikh, Kuwait; 4 School of Computing and Engineering, Faculty of Media, Science and Technology, Bournemouth University, Poole, Dorset BH12 5BB UK; 5 Departments of Aerospace and Mechanical Engineering and Materials Science, University of Southern California, Los Angeles, CA 90089-1453 USA

## Abstract

High-pressure torsion (HPT) was employed to investigate the effects of monotonic and cyclic shear deformation on the microstructural evolution and hardness of commercial purity molybdenum processed at room temperature (RT). Disks were subjected to monotonic HPT (m-HPT) and cyclic HPT (c-HPT) with strain reversals for total numbers of turns ranging from 1/2 to 7 under an applied pressure, P, of 6.0 GPa. Electron backscatter diffraction analysis showed that m-HPT promotes progressive grain refinement and continuous transformation of low-angle boundaries (LABs) into high-angle boundaries (HABs) giving an equiaxed ultrafine-grained structure with an average grain size of ~ 0.19 µm and a high fraction of ~ 82% of HABs after 7 turns. In c-HPT, there are elongated or lamellar grain morphologies, reduced grain refinement to ~ 0.25 µm and a suppressed development of HABs to ~ 67% after 7 turns. There was hardening and radial hardness homogeneity in m-HPT but slower hardening and persistent radial hardness gradients in c-HPT. It is concluded that strain reversals during c-HPT enhance a stress-assisted dynamic recovery, promote dislocation annihilation, and disrupt subgrain rotation, thereby suppressing the continuous dynamic recrystallization.

## Introduction

Processing by high-pressure torsion (HPT) involves placing a specimen, usually in the form of a thin disk, between two massive anvils, applying a high pressure and then concurrent torsional shear to impose plastic deformation [1]. Accordingly, HPT is classified as a continuous process as the specimen remains between the anvils throughout deformation and this is a condition that inherently limits the evolution of different processing routes. This contrasts with equal-channel angular pressing (ECAP) in which a rod or bar is pressed through a die composed of intersecting channels that meet at a sharp angle [2] where this is a discontinuous procedure and the specimen can be rotated around different angles between each separate passage through the die [3, 4].

Both HPT and ECAP are processing methods that involve the application of severe plastic deformation (SPD) to introduce very high shear strains without incurring any significant changes in the overall dimensions of the samples. These and other SPD processing routes have attracted much attention over the last three decades because they provide the opportunity to achieve exceptional grain refinement, producing grains that are in the submicrometer range and often within the nanometer range. Consequently, processing by SPD has been the subject of several recent reviews [5–12].

In conventional HPT, the combination of high pressure and torsion prevents cracking and allows the material to accumulate extremely large strains [13]. Ultimately, this leads to significant grain refinement and the formation of ultrafine or nanocrystalline structures. In practice, the imposed deformation varies across the disk, and the equivalent von Mises strain, ε_eq,_ at any position within the disk is given by the relationship [14]:

1 $$_{{{\epsilon_\mathrm{eq}} }} = \frac{2 \pi r N}{{\sqrt 3 h}}$$

where *r* is the radius of the disk, *N* is the total number of turns, and *h* is the thickness of the disk. Thus, the strain increases outward from the center of the disk to the periphery.

In HPT processing, it is possible to change the direction of rotation by stopping the anvil and then starting again in the opposite direction, where this can be done almost instantaneously without removing the pressure from the sample. This so-called strain reversal testing was first introduced in 2005 in early HPT experiments on an Al–Mg–Sc alloy [15], and it provided the potential for developing two different processing procedures in HPT, termed either monotonic HPT (m-HPT) where processing is continuous in the same direction or cyclic HPT (c-HPT) where the direction of rotation is abruptly reversed during the processing operation. Thus, the use of c-HPT opens the possibility of investigating the effect of the strain path on the microstructures and the subsequent mechanical properties of the material.

Several investigations have been conducted to evaluate the differences in processing by m-HPT and c-HPT, and data are now available for tests conducted in combinations of monotonic and cyclic testing of pure Al [16–21], Al alloys [22–24], pure Cu [25], pure Ni [26, 27], Armco iron [26], steels [28], pure Ti [29], Zn-22% Al [30], Ti-6Al-4 V [31, 32], and a CoCuFeMnNi high entropy alloy [33]. An examination of the evolution of hardness in these various reports shows that processing by m-HPT generally leads to a rapid hardness increase during the initial stage of HPT straining, followed by a steady-state saturation hardness as the ultrafine-grained (UFG) microstructure develops. Such hardness saturation is governed by the limit of grain refinement, typically in the range of ~ 50–200 nm, due to saturation of the grain boundary misorientations and the development of a balance between strain hardening and dynamic recovery. By contrast, the periodic reversal of shear during c-HPT disrupts the dislocation structures and thereby reduces the formation of stable cell structures and the subsequent subgrain formation, which is a prerequisite for achieving a UFG microstructure. Consequently, hardness typically evolves to a higher saturation strain level in c-HPT compared to m-HPT. In general, a higher rate of increase in the values of the hardness using m-HPT is anticipated compared with disks processed by c-HPT, but there was an exception with pure Cu [25] where the hardness values were highest in c-HPT.

Processing by HPT has generally focused on metals having face-centered cubic (FCC) crystal structures, such as Al [34], Cu [35, 36], Ni [37], and Ag [38] or hexagonal close-packed (HCP) crystal structures such as Mg [39], Ti [40], Zn [41], and Zr [42]. Fewer investigations were devoted to metals having body-centered cubic (BCC) crystal structures, and most of these investigations were conducted on iron and carbon steels [43]. Compared to FCC metals of low stacking fault energy (SFE), BCC metals have a greater tendency for dislocation cross-slip and climb. This promotes dynamic recovery during HPT straining, leading to annihilation of dislocation tangles, typically seen as free isolated dislocations within cells or subgrains, while allowing the remaining dislocations to reorganize into cells and/or subgrains. These subgrains can then rotate with increasing strain into ultrafine grains with grain boundaries having high angles of misorientation. Such grain refinement in BCC structures was found to occur primarily by continuous dynamic recrystallization (cDRX) [44], but the continuous removal of excess dislocations by dynamic recovery limits the amount of grain refinement and leads to saturation at a relatively larger grain size. In addition, dynamic recovery reduces the retained dislocation density within the UFG structure, limiting dislocation storage and thereby reducing the hardening response during severe plastic deformation.

## HPT processing of molybdenum and other refractory metals

The refractory metals such as Mo, Ta, W, and Nb have a BCC structure and they are hard-to-deform metals having exceptionally high melting points. For example, Mo melts at 2610 °C (2883 K) and Nb melts at 2477 °C (2750 K) and these values are approximately double the value for iron. This means, therefore, that the homologous temperature (*T*/*T*_m_, where *T*_m_ is the absolute melting temperature) is ~ 0.1 for refractory metals at room temperature which is far below the typical recrystallization temperature of ~ 0.25*T*_m_ for pure metals. Consequently, it is anticipated that HPT processing will be an efficient method for the development of ultrafine grain refinement in refractory metals [45]. Additionally, these metals possess excellent mechanical stability at high temperatures and a resistance to softening.

In practice, the refractory metals are important as potential materials for use in many applications. For example, pure Mo and its alloys exhibit several distinguishing properties including high creep resistance, high stability, and high thermal conductivity, and this makes them attractive for use as electrodes in furnaces, as corrosion-resistant coatings, and as alloying agents for other metals [46, 47]. Molybdenum also has potential for use as a biodegradable implant material for structural applications [48]. Furthermore, it is important to note that there is now an increasing interest in processing refractory ceramic materials by HPT [49].

Conventional hot compression of pure Mo in the temperature range of 1000–1500 °C (approximately 0.44–0.61 *T*_m_) was reported to exhibit recovery-controlled deformation behavior due to the high mobility of dislocations and the strong tendency for recovery in molybdenum [50]. The predominance of dynamic recovery within this temperature range was also confirmed in another report but at higher temperatures of 1600–1700 °C (approximately 0.66–0.70 *T*_m_); there was dynamic recrystallization accompanied by grain growth [51]. By contrast, during HPT processing of refractory BCC metals such as Mo, dynamic recovery and continuous dynamic recrystallization (cDRX) may occur even at very low homologous temperatures. Under these conditions, the severe plastic deformation generates exceptionally high stored strain energy, extremely high defect densities and large hydrostatic pressures. These conditions together promote stress‑assisted dislocation rearrangement and progressive subgrain formation, enabling the gradual development of low‑angle boundaries without requiring significant thermal activation. Consequently, the deformation mechanisms are anticipated to be driven primarily by significant mechanical energy and strain accumulation rather than by the diffusion‑controlled processes which are typical of high temperature deformation [52].

There have been very few reports to date of monotonic HPT investigations of Mo [53–59]. In an early report, monotonic HPT was conducted on high-purity Mo at ambient temperature under applied pressures of 2 to 6 GPa for up to 10 turns and the results showed an increase in the Vickers microhardness with increasing pressure to reach a maximum value of ~ 680 Hv under a pressure of 6 GPa with an average grain size of ~ 350 nm [53]. It was suggested that HPT conditions of 6 GPa promote substantially greater dislocation storage together with more intense stress‑driven annihilation, thereby producing a distinct microstructural evolution that reaches saturation at an earlier strain level. Another investigation recorded hardness measurements in Mo samples together with many other pure FCC, HCP, and BCC metals after processing by monotonic HPT, and the results demonstrated that the hardness of Mo increased following HPT processing and reached an ultimate stable value of approximately ~ 680 Hv [54]. A study of commercial purity (CP) molybdenum (99.95%) by ECAP for 1 and 2 passes and by monotonic HPT for 5 and 10 turns demonstrated that HPT achieved finer grain sizes and higher microhardness values than ECAP [60]. In an investigation of CP molybdenum, disks were processed by HPT at room temperature for 1/2 to 10 turns under a pressure of 6.0 GPa; the average grain size decreased from 360 μm in the initial annealed condition to about 690 nm after 5 turns, while the microhardness increased from 194 Hv to about 660 Hv after HPT for 5 turns [61]. However, the microhardness decreased slightly after 10 HPT turns due, it was proposed, to limited dynamic recovery. There are also reports describing the properties of other refractory metals such as V, Ta, Nb, and W after HPT processing [53, 62–70].

Nevertheless, all of these studies on the HPT processing of refractory metals have focused on conventional monotonic HPT to produce ultrafine-grained structures and enhanced hardness. Accordingly, the present investigation was conducted to provide, using CP Mo, the first direct comparison between monotonic and cyclic HPT for a representative refractory metal. Since BCC refractory metals such as pure Mo and Ta are known to exhibit a modest level of dynamic recovery under HPT deformation at low homologous temperatures due to the large stored strain energy and high dislocation density [61, 63, 71], it is reasonable to anticipate that such processes may influence the minimum achievable grain size and the retained dislocation density. To perform these experiments, detailed characterizations of the microstructure and the microhardness evolution were conducted using scanning electron microscopy (SEM) and electron backscattering diffraction (EBSD) combined with direct measurements of the Vickers microhardness.

## Experimental material and procedures

The experiments were conducted using CP (99.95%) molybdenum having a chemical composition as shown in Table 1. The material was supplied as a long circular bar with a diameter of 10.0 mm, and disks were cut from the bar for HPT processing with thicknesses of ~ 1.6 mm using brass wire erosion with an electro-discharge machine. Prior to testing, the thickness of each disk was reduced to 0.8 mm by mechanical polishing.

**Table 1:** Chemical composition of CP Mo (in ppm)

Element	Al	Cr	Cu	Fe	K	Ni	Pb	Cd	C	H	N	O	Si	W
Amount	10	< 20	< 20	< 20	< 20	< 10	< 5	< 5	< 30	< 10	< 10	< 40	< 20	< 300

The HPT processing was conducted in air at RT with a speed of rotation of 1 rpm in both directions, an applied pressure, *P*, of 6.0 GPa and with the processing conducted under quasi-constrained conditions where there is a limited outflow of material around the periphery of the disk during processing [72, 73]. This outflow produces a slight reduction in the disk thickness during HPT. The disks were divided into two separate sets and Table 2 summarizes the numbers of turns applied to the two sets. For the first set in m-HPT, the forward direction was used to strain the disks monotonically for totals of 1/2, 1, 5, and 7 turns, respectively. For the second set in c-HPT, the rotation was terminated and the straining direction was reversed during processing with the strain reversal completed within two seconds. The c-HPT samples were subjected to totals of 1/2, 1, 5, and 7 turns where routes A and B in Table 2 denote rotations in the clockwise (CW) and counter-clockwise (CCW) directions, respectively.

**Table 2:** Total numbers of HPT turns

Total number of turns	m-HPT	c-HPT
N = 1/2	1/2	1/4A + 1/4B
N = 1	1	1/4A + 1/4B + 1/4A + 1/4B
N = 5	5	1A + 1B + 1A + 1B + 1A
N = 7	7	1A + 1B + 1A + 1B + 1A + 1B + 1A

After HPT processing, the disks were mounted and ground on the radial direction-tangential direction (RD-TD) plane using 120, 600, 1200, and 4000 grit SiC papers. This was followed by polishing on a metallographic cloth using 9 and 3 µm diamond pastes, respectively. Finally, the disks were prepared to mirror-like finishes by electro-polishing for microstructural evaluation by EBSD along the axial direction (AD). Micrographs for the distributions of grain sizes and the misorientation angles of all disks at positions near the edges, corresponding to the highest imposed strains, were taken using an HKL Channel-5 EBSD detector interfaced to a JEOL F7001 field-emission SEM operating at 20 kV. The EBSD maps were constructed so that thin gray lines represent low-angle boundaries (LABs) with misorientation angles below 15° and high-angle boundaries (HABs) with misorientation angles above 15° are shown as solid black lines. A micrograph of the sample was also recorded in the as-received and unprocessed condition.

A potential problem in HPT is that slippage may occur during the processing operation, where slippage is defined as the difference between the torsional rotation measured in the disk and the rotation imposed externally using HPT. Earlier measurements on different metals showed that the extent of slippage is dependent upon the surface condition of the anvils in the HPT facility, and in practice slippage increases both at faster rotational speeds and at lower imposed pressures [74]. In the present experiments, initial tests were conducted using a pressure of 6.0 GPa at room temperature with new anvils in the HPT facility and with parallel lines scribed on the upper and lower surfaces of the disks. Measurements of the angles between these lines after processing showed there was no significant slippage in these experiments under either monotonic or cyclic conditions.

A second potential problem concerns the temperature rise that may occur in the specimen during the processing operation. There have been several investigations designed to measure the temperature rise in various metals processed by HPT [73, 75–78], and the overall results from these studies are consistent by showing that the temperature rise in HPT is of only minor significance. Furthermore, although the increase in temperature is directly proportional to the hardness of the material and Mo is a hard material, earlier experiments on Mo demonstrated conclusively that the temperature increase in Mo when processing by HPT at RT was of little or no significance compared with the low absolute melting temperature of the material.

The Vickers microhardness, Hv, was measured along diameters of the disks using an InnovaTest Vickers microhardness tester by applying a load of 300 gf with a dwell time of 15 s. These hardness measurements were taken on each disk along four diameters arranged at angles of 45^°^ and with a total of 33 measurements, spaced 0.3 mm apart, recorded along each diameter. The microhardness results after processing by m-HPT and c-HPT were then plotted as the average microhardness value against the position along the disk diameter. The average value of Hv was also recorded in the as-received unprocessed condition. Finally, the average microhardness measurements were plotted against the equivalent von Mises strain, ε_eq_, estimated from Eq. (1).

## Experimental results

### Microstructural observations

**Figure 1:**
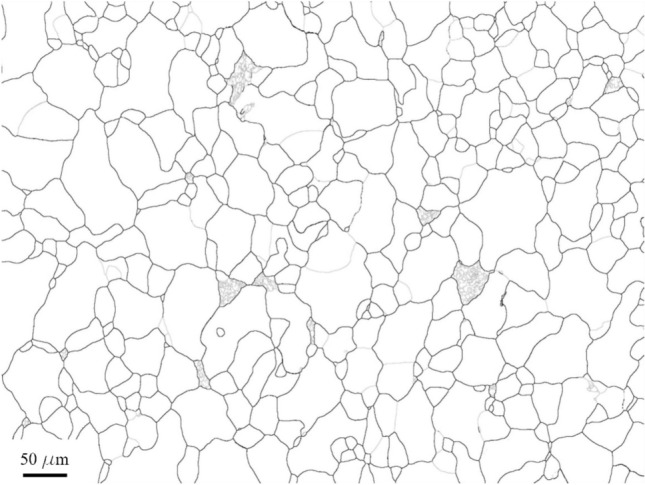
Microstructure of CP Mo in the as-received unprocessed condition.

The microstructure of the as-received unprocessed molybdenum is shown in Fig. 1, and it consisted of well-defined equiaxed grains, primarily having HABs, with an average grain size of ~ 26 μm. Subgrain and dislocation cell structures, delineated by LABs, also exist in a few grains. This variation in the microstructure is feasible in BCC metals when the local stored energy is insufficient to cause recrystallization, and/or there is recovery favoring polygonization of the dislocation cell structure [79].

**Figure 2:**
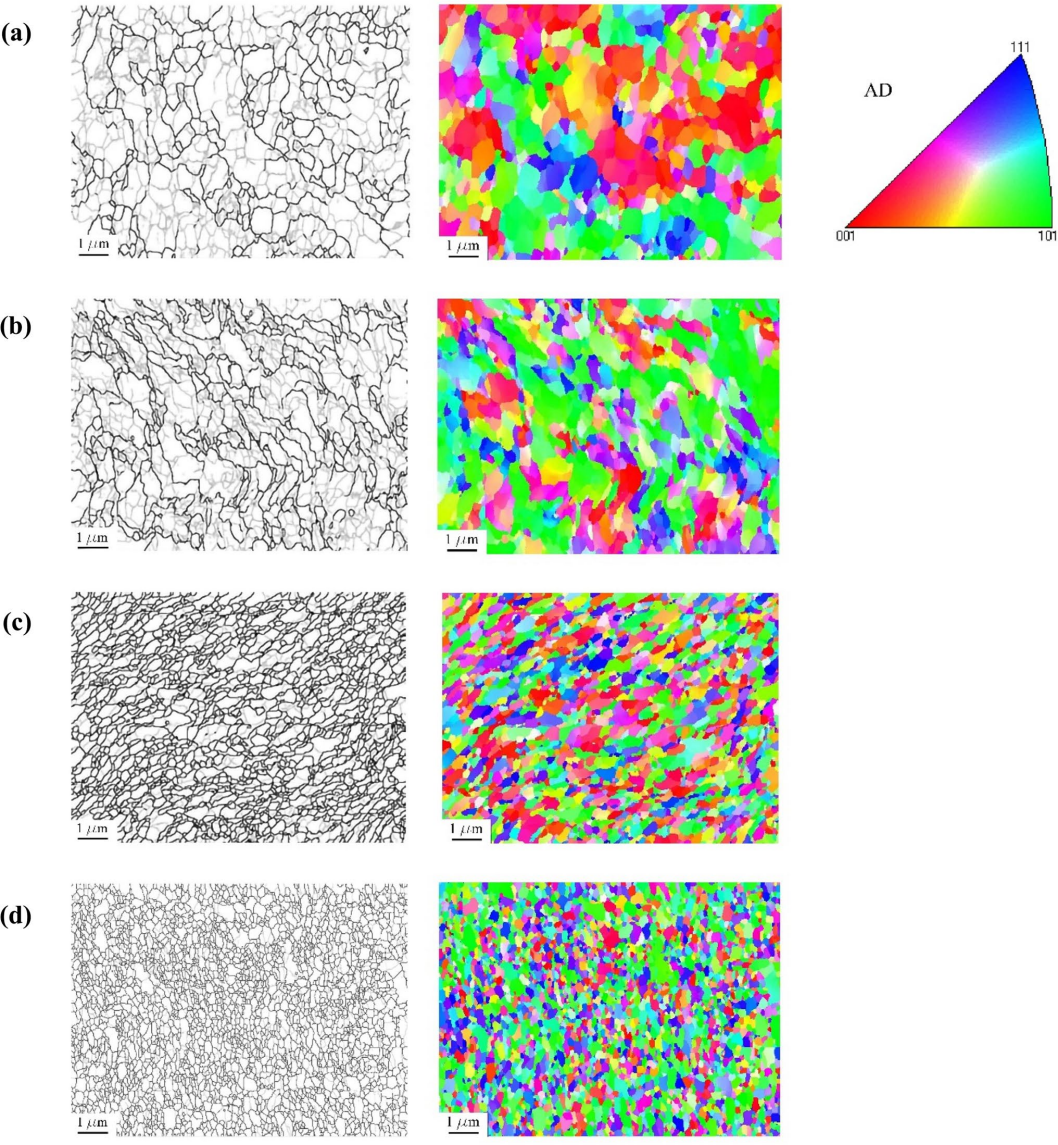
Images near the edges of disks processed by m-HPT for **a** 1/2, **b** 1, **c** 5, and **d** 7 turns: the images in the left column show individual grain boundaries within each area where the HABs are marked in bold black and the LABs are marked in light gray and the images in the right column show the inverse pole figure maps (AD: Axial direction).

**Figure 3:**
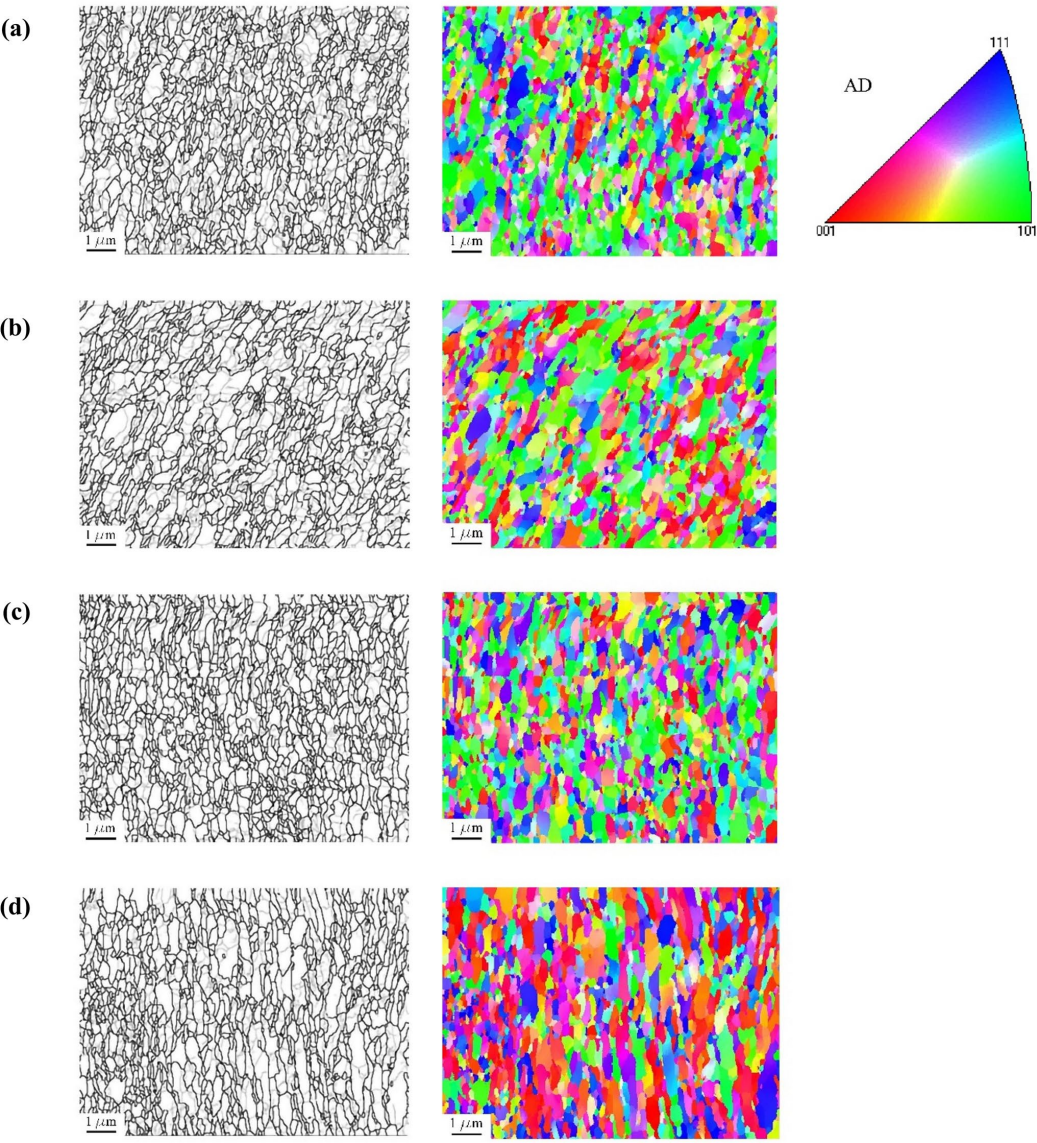
Images near the edges of disks processed by c-HPT for **a** 1/2, **b** 1, **c** 5, and **d** 7 turns: the images in the left column show individual grain boundaries within each area where the HABs are marked in bold black and the LABs are marked in light gray and the images in the right column show the inverse pole figure maps (AD: Axial direction).

Figure 2 shows the near-edge positions of the disks after m-HPT through 1/2, 1, 5, and 7 turns, where the images on the left show individual grain boundaries within each area with the HABs marked in bold black and the LABs marked in light gray and the images on the right are the inverse pole figure (IPF) maps. A similar set of illustrations is given in Fig. 3 for the c-HPT condition. After processing at low strains of 1/2 and 1 turn in m-HPT, as shown in Fig. 2 (a and b), the microstructure consisted of coarse and slightly elongated grains having a mixture of HABs and LABs with a higher fraction of LABs. However, after 5 and 7 turns of m-HPT in Fig. 2 (c and d), there was significant grain refinement and most of the grains had HABs. A general feature of the microstructure after c-HPT was that most grains were elongated for all numbers of turns and at 1/2 and 1 turn in Fig. 3 (a and b); there was a stronger development of HABs than in the comparable m-HPT samples. Furthermore, the grains were coarser after 1/2 and 1 turn in c-HPT but increasing the numbers of turns to 5 and 7 for c-HPT in Fig. 3 (c and d) produced no significant effect on the fraction of HABs. Grain coarsening was clearly visible in many of the grains of the sample processed by 7 turns in c-HPT but this coarsening was not apparent in the sample processed by m-HPT.

**Figure 4:**
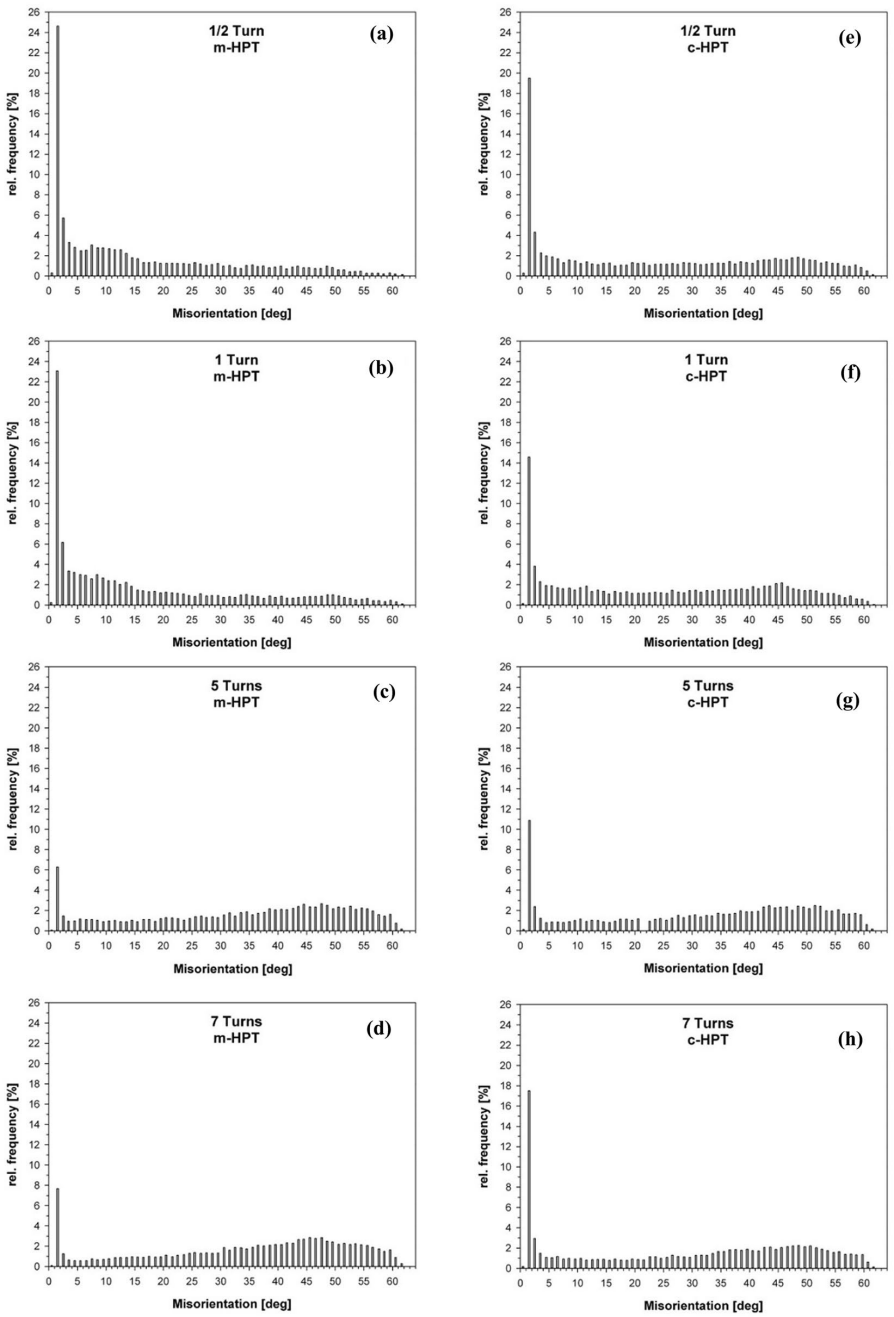
Distribution of the number fraction of the misorientation angles of the grain boundaries of the disks processed by m-HPT and c-HPT for 1/2, 1, 5, and 7 turns.

Further analyses are shown in Fig. 4 where the distributions of the misorientation angles are depicted for disks processed by m-HPT (left column) and c-HPT (right column) for totals of 1/2 to 7 turns. For the disks processed by m-HPT for 1/2 and 1 turn, most of the grain boundaries were LABs but after 5 and 7 turns it is apparent that many of the LABs transformed into HABs. For c-HPT processed through 1/2 and 1 turn, the results are similar to the m-HPT samples but with a slight increase in the fraction of HABs, and after 5 and 7 turns the distributions show fewer HABs than in the m-HPT samples.

The average grain sizes and the length fractions of the HABs near the edges of the disks processed by m-HPT and c-HPT are summarized in Table 3. These measurements indicate minimum grain sizes of ~ 0.23 µm after 1 and 5 turns in m-HPT and ~ 0.22 µm after 1/2 turn in c-HPT. Achieving a minimum grain size at this low strain in c-HPT suggests that there is a transient refinement effect associated with the strain reversal. The highest fraction of HABs was recorded at 82% after m-HPT through 7 turns and this contrasts with a fraction of 67% after 7 turns in c-HPT. This behavior demonstrates there is a suppressed conversion of LABs into HABs at high strains in c-HPT, and this effectively limits the development of a stable ultrafine‑grained structure.

**Table 3:** EBSD grain size and length fraction of HABs near the edges of CP Mo disks

	m-HPT		c-HPT	
	Grain size (µm)	Fraction of HABs (%)	Grain size (µm)	Fraction of HABs (%)
N = ½	0.27	40	0.22	59
N = 1	0.23	41	0.24	62
N = 5	0.23	80	0.24	75
N = 7	0.19	82	0.25	67

### Microhardness measurements

**Figure 5:**
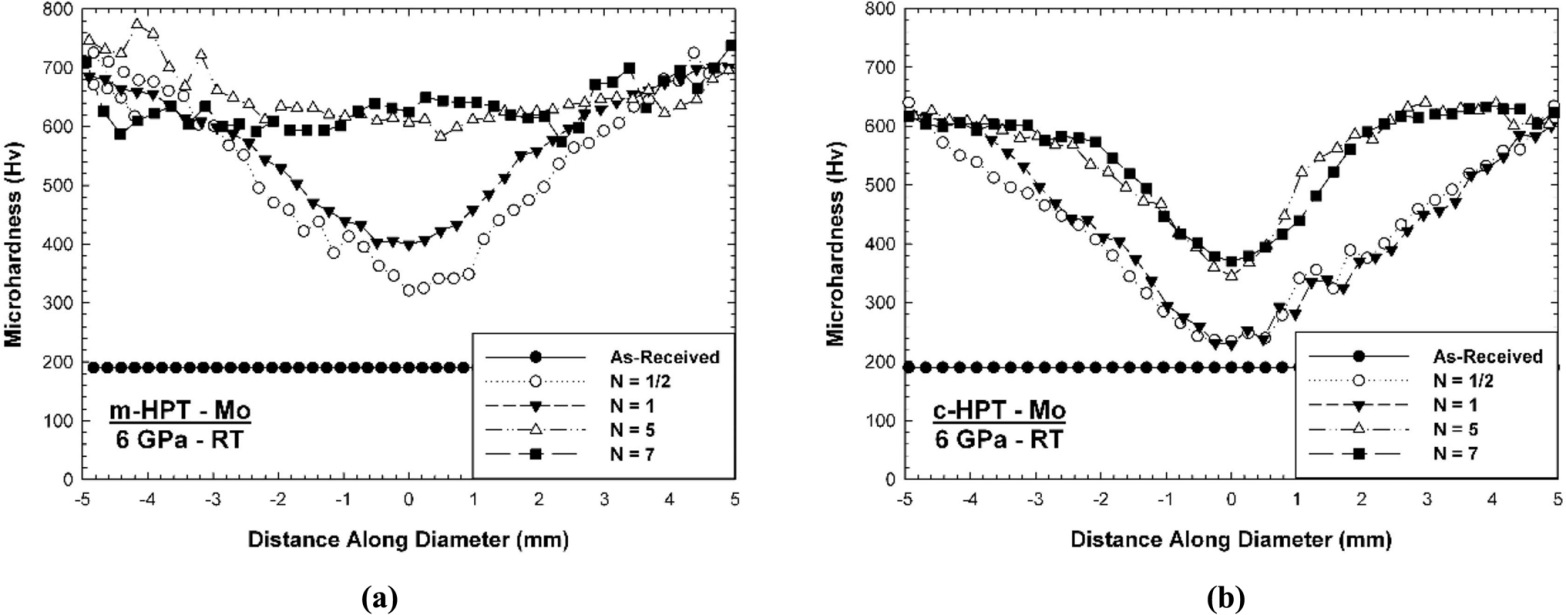
The average microhardness Hv recorded along the diameters of molybdenum disks after **a** m-HPT and **b** c-HPT for 1/2, 1, 5, and 7 turns: the average microhardness in the as-received unprocessed condition is also shown.

Figure 5 compares the average Vickers microhardness measurements across the diameters of the disks in (a) m-HPT and (b) c-HPT, respectively, together with the average measurements of Hv in the as-received condition where the hardness was ~ 190 Hv. For both the m-HPT and c-HPT conditions, the microhardness after 1/2 and 1 turn is higher than for the as-received material and the values of Hv then increase reasonably linearly to reach maximum values at the outer edges. In addition, the microhardness values after m-HPT for 5 and 7 turns become fairly constant along the diameters of the disk with values in the range of ~ 600–640 Hv and slight increases to ~ 740 Hv at the edges. No similar constant values of Hv are visible in the c-HPT sample after 5 and 7 turns, and there are again lower values in the central region increasing to values of ~ 620 Hv near the outer edges.

For each testing condition, the m-HPT disk has higher values of Hv than the c-HPT disk, where this confirms the earliest result reported for monotonic and cyclic testing using an Al–Mg-Sc alloy processed through either 2 forward turns or 1 forward turn and 1 reverse turn [15]. Nevertheless, a full interpretation of the present experimental results requires a comprehensive comparison with other published data, and this is described in the following sections.

## Discussion

### Comparison of microstructure and hardness for m-HPT and c-HPT

The microstructural evaluation of the m-HPT and c-HPT processing conditions indicates that, with increasing numbers of turns from 1/2 to 7, the m-HPT samples exhibit a greater tendency for the formation of equiaxed grains and this is accompanied by a more enhanced grain refinement, from 0.27 µm after 1/2 turn to 0.19 µm after 7 turns, and with a significantly increasing fraction of HABs from 40 to 82%, respectively. This progressive refinement of grain size near the edges of the disks with increasing numbers of turns is consistent with the increasing fraction of HABs, and it demonstrates the general stability of the microstructure. By contrast, increasing the numbers of turns in c-HPT produces less grain refinement at the edge, from 0.22 µm after 1/2 turn to 0.25 µm after 7 turns, and this is accompanied by the development of an elongated or lamellar grain morphology rather than a fully equiaxed structure. This microstructural development in c-HPT also leads to a slower evolution in the fraction of HABs from 59% after 1/2 turn to 67% after 7 turns. The use of c-HPT in these experiments also suggests the occurrence of a microstructural instability since the finest grain size was achieved after processing for 1/2 turn and the average grain size thereafter gradually increased with increasing numbers of turns. This instability is confirmed by the evolution of HABs because the maximum fraction of 75% was attained after 5 turns and there was a decrease to 67% after 7 turns.

In addition, a detailed inspection shows that processing by c-HPT produces different microstructural developments depending upon the magnitude of the strain reversal. Thus, if there is a small increment of strain reversal, as when using 1/4 turn in the c-HPT samples processed by 1/2 and 1 turn, there is then a greater enhancement in the grain refinement and less development of the HABs fraction. Conversely, when using a higher increment of strain reversal, as when using 1 turn for the c-HPT samples processed by 5 and 7 turns, the refinement of the grains is less and the fractions of HABs are slightly higher. This result demonstrates that a large increment of strain reversal tends to increase the dislocation annihilation per cycle and thereby slow the grain refinement process. Similar results were evident earlier in the processing of high-purity aluminum [20] where, using c-HPT for 4 turns with 1 turn each cycle, there was a higher hardness compared with using a larger increment of straining, such as 2 turns per cycle, for the same total numbers of turns. This effect is attributed to a reduction in the annihilation of dislocations during processing by c-HPT when using smaller increments of straining.

In m-HPT, the shear strain accumulates continuously in one direction and this promotes a progressive grain subdivision into dislocation cells and subgrains that steadily rotate and increase their misorientations [1, 80, 81]. This uninterrupted buildup of misorientation allows the LABs to transform relatively rapidly into HABs through cDRX, ultimately producing an equiaxed UFG structure and the early development of a stable HAB-rich structure [82]. By contrast, c-HPT imposes repeated reversals of the shear direction and this serves to partially annihilate the accumulated dislocations and to interrupt the stable subgrain rotation process that is needed for the conversion of LABs into HABs. This increase in the annihilation of dislocations during c-HPT is attributed to the dynamic recovery of molybdenum, and similar observations were recorded also in FCC metals with high SFE such as aluminum [20]. Nevertheless, it should be noted that the description of dynamic recovery has been refined to emphasize that, in BCC refractory metals deformed at low homologous temperatures, recovery-like behavior arises primarily from stress-assisted dislocation rearrangement and subgrain formation, whereas dislocation annihilation remains limited but critical in strain reversal conditions. These mechanisms are distinct from the diffusion‑controlled climb‑dominated recovery that characterizes high‑temperature deformation [52].

Several reports indicate that the strain reversals during cyclic deformation enhance dynamic recovery and suppress the buildup of large stored strain energies [18, 83, 84]. Thus, each strain reversal partially counteracts the misorientation already accumulated and thus suppresses the HAB development and leads to grain refinement at a reduced rate. As a result, c-HPT produces more elongated or lamellar grains which are less refined and with an associated smaller fraction of HABs.

### Characteristics of the grain refinement mechanisms in m-HPT and c-HPT

The difference in grain size and the characteristics of the grain boundaries indicate possible differences in the grain refinement mechanisms for the m-HPT and c-HPT processing routes. For m-HPT, the constant shearing direction leads to a steady accumulation of dislocations and a progressive rotation of subgrains which enables the LABs to transform systematically into HABs. Such microstructural development demonstrates that the primary mechanism for grain refinement is driven by cDRX. By contrast, the strain reversals during c-HPT reduce the net rate of dislocation storage through dislocation annihilation and rearrangement and, in strain reversal, dynamic recovery becomes more pronounced and this limits the lattice rotation necessary for cDRX. When the shear direction is reversed, the pre-existing dislocation substructures, such as dislocation cells, become unstable and this makes it possible for those dislocations to partially annihilate so that the dynamic recovery is accelerated, and the dislocation density is reduced. In practice, the strain reversal disrupts the accumulation of geometrically necessary dislocations (GNDs) that are a prerequisite for subgrain rotation and thereby serves to suppress cDRX. Additionally, this strain reversal can create local incompatibilities and strain gradients; it is reasonable to anticipate that, at sufficiently high strains in c-HPT, this shear localization will promote the formation of shear bands. Experimental evidence is already available for UFG Ni showing enhanced boundary migration and grain coarsening within the shear bands produced by c-HPT [85]. The general conclusion from this analysis is that grain refinement in c‑HPT will proceed at a slower rate of cDRX, and this will allow other mechanisms such as dynamic recovery and shear‑band‑assisted refinement to participate, ultimately leading to the formation of coarser and more elongated grains together with a lower fraction of HABs compared with processing by m‑HPT.

The strong grain refinement and excellent storage of dislocations in m-HPT correlates with the outstanding increase in hardness which, as shown in Fig. 5a, reaches a reasonable saturation and radial hardness uniformity of ~ 600–640 Hv after 5 turns. There is also little or no change in hardness when increasing the straining to 7 turns. This is indicative of a microstructural saturation where both the dislocation density and the grain size are at a saturation value. The overall evolution of the microstructure and microhardness of the CP Mo under monotonic HPT processing in the present investigation is generally consistent with that of some previous investigations on pure Mo [53, 54, 61]. Thus, in these earlier investigations m-HPT was conducted at room temperature under imposed pressures between 2 and 6 GPa for numbers of turns ranging between 1/4 and 10. The results from these investigations showed an increase in the microhardness measurements to a maximum saturation value of approximately 680 Hv and a decrease in the average grain size to a value of ~ 0.35 µm under the highest pressure and largest numbers of turns.

In the use of c-HPT, the repeated reversals of the shear direction lead to a partial dislocation annihilation and rearrangement, and this promotes dynamic recovery and reduces the overall dislocation accumulation. As a result, the hardness evolves more slowly in c-HPT without achieving radial uniformity, so that lower peak hardness values are attained at the disk edge compared to those in m-HPT for the same numbers of turns. In m-HPT, the strain accumulates continuously allowing the regions of lower strain near the center to gradually reach a saturation state and thus exhibit a relatively uniform hardness. By contrast, the strain reversals in c-HPT partially cancel the strain more efficiently in the low strain regions so that the dislocation structures in the early stages are destabilized, and this means the strain reversals disrupt the subgrain rotation and accelerate recovery in the high strain regions near the disk edges. Therefore, this inhomogeneity in c-HPT leads to a reduced uniformity in hardness across the disk.

### Dependence of microhardness on strain

**Figure 6:**
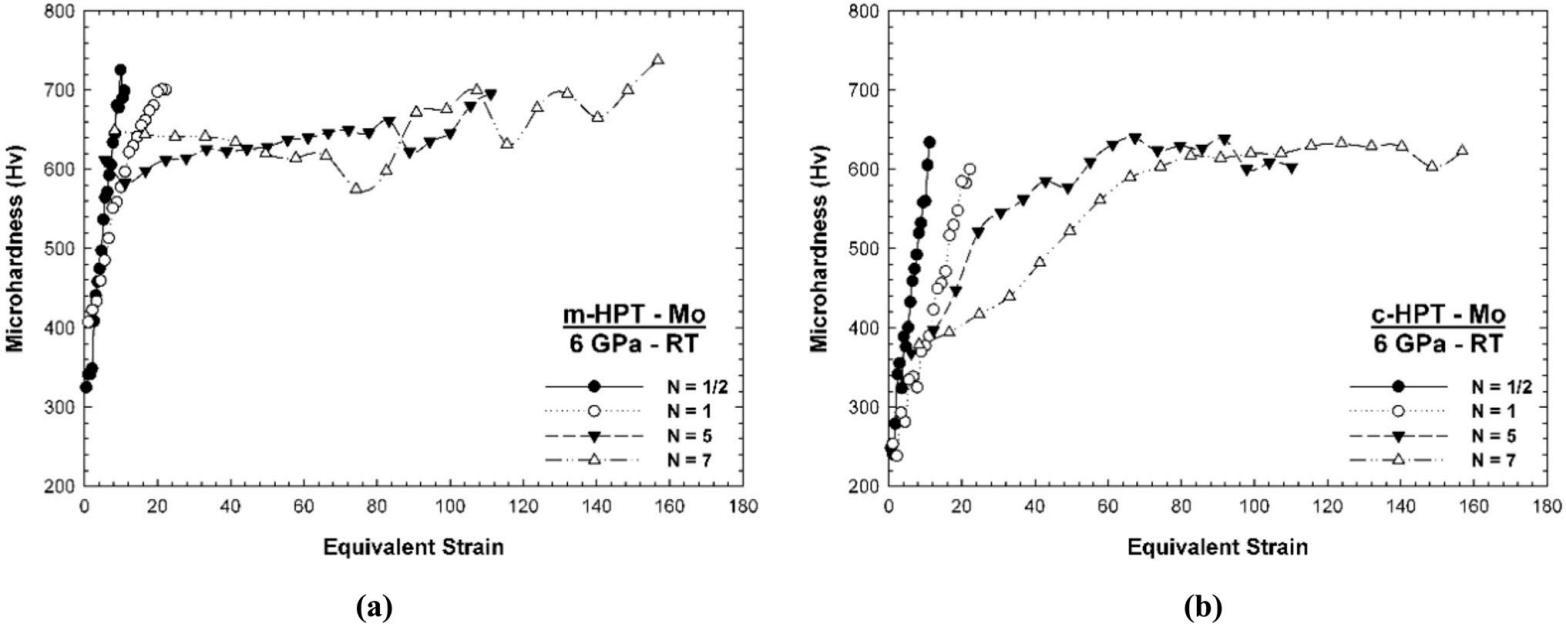
The average microhardness versus the equivalent strain for Mo disks after **a** m-HPT and **b** c-HPT.

To more closely examine the evolution of Hv with increasing strain, it is important to follow an earlier proposal for the interpretation of hardness measurements in HPT [86], where the hardness measurements were plotted against the local equivalent von Mises strain, ε_eq,_ calculated at every measurement point along the radius of the disk. These plots are shown in Fig. 6 for the Mo samples tested in (a) m-HPT and (b) c-HPT using the same datum points already plotted in Fig. 5. The plots reveal an overall hardening with increasing strain in both processing conditions, but it is also apparent that a saturation hardness is attained more rapidly when processing by m-HPT. Thus, a saturation hardness of ~ 600 – 650 Hv was reached in m-HPT after an imposed strain of ε_eq_ ≈ 10, whereas in c-HPT the saturation hardness was delayed to strains above ε_eq_ ≈ 70.

To properly assess the significance of the hardness measurements, it is important to follow earlier reports and consider the different types of hardness evolution that may result from HPT processing based on the nature of the predicted curves for Hv against ε_eq_ [30, 87, 88]. It was proposed in early experiments on the monotonic and cyclic testing of a two-phase Zn-22% Al eutectoid alloy that there are three possible types of hardness evolution [30]. The first type is termed hardening without recovery where the hardness simply increases with strain and then stabilizes and remains constant at a saturation value. Examples of this type of behavior include CP Al [89–91], Cu [92, 93], Fe [94, 95], and Ti [96]. The second type of evolution is termed hardening with recovery where the hardness first increases gradually with increasing strain, reaches a maximum and then decreases to a steady-state value where the hardness remains constant with increasing strain but with a value which is lower than the peak hardness but higher than the initial hardness. This type of behavior occurs in materials with high SFE such as high-purity Al [16, 87, 93, 97, 98], high-purity Zn [87], and Mg [99]. The third type of evolution is termed weakening where the hardness decreases gradually with increasing strain and reaches a steady-state value which is either slightly lower or significantly lower than in the initial condition.

Considering this third type, the former effect of a slightly lower hardness occurs in materials such as Pb [87], Sn [87], and In [87] where processing by HPT at room temperature corresponds to a high homologous temperature; the average grain sizes produced by HPT are of the order of ~ 100 µm or larger. Additional experiments showed that the hardness is slightly lower than in the initial condition in these materials because of a breakdown in the Hall–Petch relationship at high homologous temperatures [100] where this is associated with a contribution from grain boundary effects such as grain boundary sliding [101] that effectively reduces the significance of strain hardening. The second effect of a much lower hardness occurs in two-phase alloys such as the Zn-22% Al eutectoid alloy [30, 102], the Pb-62% Sn eutectic alloy [102], and the Bi-42% Sn eutectic alloy [103], where the final hardness is significantly lower than in the initial condition due to a loss of precipitates during the HPT processing. A comprehensive description of the effects of strain hardening and strain weakening in SPD processing was presented earlier [104]. Based on these well-developed types of hardening, it is readily apparent from Fig. 6 that the CP Mo used in the present experiments exhibits hardening but with an overall level of recovery which is insufficient to introduce measurable softening at least up to the strain induced by HPT through 7 turns. This means, therefore, that molybdenum is similar to most other metals by exhibiting strain hardening in the initial stages of HPT processing.

## Summary and conclusions

1. Disks of commercial purity molybdenum were processed by HPT both monotonically (m-HPT) and cyclically (c-HPT) at room temperature for totals of 1/2, 1, 5, and 7 turns under an applied pressure of 6.0 GPa.
2. The disks processed by m‑HPT showed a stronger tendency to form equiaxed grains, with pronounced grain refinement to 0.19 µm and a high fraction of 82% HABs after 7 turns. The continuous, unidirectional shear strain in m‑HPT promotes progressive grain subdivision and increasing misorientation, enabling LABs to transform into HABs through cDRX. This is accompanied by a significant hardness increase that reaches saturation and a radial uniformity at large strains.
3. In c‑HPT, there is less grain refinement to 0.25 µm after 7 turns and an elongated or lamellar grain morphology with a lower fraction of 67% HABs. The repeated strain reversals partially annihilate the accumulated dislocations and disrupt the transformation of LABs to HABs. The hardness increases more gradually in c‑HPT without achieving radial uniformity, and the peak values at the disk edges are lower than those achieved by m‑HPT for the same numbers of HPT turns.
4. The results of the microhardness measurements demonstrate a hardening without significant recovery in both m-HPT and c-HPT, but the saturation hardness can be attained at a lower strain when processing by m-HPT.

## Data Availability

Data will be made available upon reasonable request.
